# Two-port approached thoracoscopic carina reconstruction using natural bronchial bifurcation

**DOI:** 10.1186/s13019-016-0541-9

**Published:** 2016-10-18

**Authors:** Tong Qiu, Yandong Zhao, Jianfang Song, Bo Fu, Yunpeng Xuan, Wenjie Jiao

**Affiliations:** 1 Department of Thoracic Surgery, The Affiliated Hospital of Qingdao University, Jiangsu Lu 16, Qingdao, China; 2 Department of Anesthesia, The Affiliated Hospital of Qingdao University, Qingdao, China

**Keywords:** Lung cancer surgery, Tracheal carina, Mediastinal lymph nodes, Minimally invasive surgery, Rapid prototyping

## Abstract

**Background:**

Carina resection and reconstruction is a challenging procedure for thoracic surgeons. We describe a novel technique of thoracoscopic carina reconstruction using the natural bifurcation, following pulmonary resection of the lung neoplasm. To our knowledge, it is the first report of this kind.

**Case presentation:**

A 71-year-old male diagnosed of squamous cell lung cancer received two-port approached video-assisted thoracoscopic right bilobectomy with carina resection after 2 cycles of neoadjuvant therapy. After the removal of right lower lobe and middle lobe, the 7 station lymph nodes were resected with the invaded carina and bronchial walls in an en-bloc fashion. The neocarina was reconstructed by the natural bifurcation between the right upper bronchus and the bronchus intermedius. Intraoperative blood loss was about 220 mL, and operative time was about 225 min. The postoperative course was uneventful. The pathological TNM stage was pT3N2M0, IIIA. Adjuvant chemotherapy using gemcitabine and cisplatin was administered for 4 cycles. Follow-up 6 months after surgery confirmed no stenosis and no signs of local recurrence by bronchoscopy and CT scan.

**Conclusions:**

We consider that the surgical procedure described here is a new alternative strategy for carina resection and reconstruction in the similar situation. The minimally invasive method is safe and effective for this challenging operation.

**Electronic supplementary material:**

The online version of this article (doi:10.1186/s13019-016-0541-9) contains supplementary material, which is available to authorized users.

## Background

Carina resection and reconstruction is a challenging procedure for thoracic surgeons. The surgical method is widely dependent on the location and extent of the invasion. Here we present a minimally invasive surgical technique to perform this procedure by using the natural bronchial bifurcation for the neocarina. To our knowledge, it is the first report of this kind.

## Case presentation

**Fig. 1:**
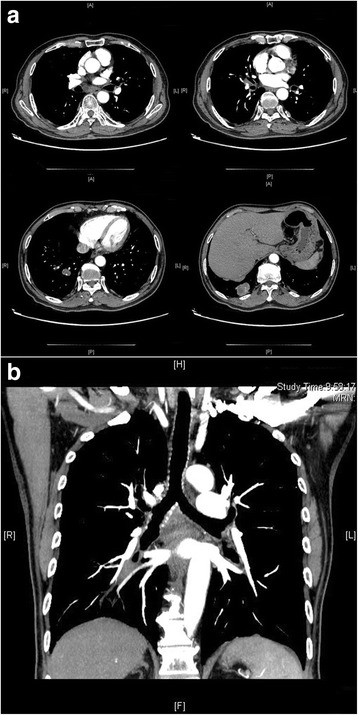
**a** Series of CT images showed two lesions in *right lower* lobe with enlarged subcarinal, *right* hilar and interlobar lymph nodes. **b** Coronal CT image showed the relationship between the subcarinal lymph nodes and the airways

A 71-year-old male presented with 2 months of cough and dyspnea. He had a 50-year smoking history and 1 month of smoking cessation. Physical examination was unremarkable. The position emission tomography and CT scan revealed two pieces of connected neoplasm located in the right lower lobe, and local lymph nodes metastases in the 7, 10R and 11R stations (Fig. 1). No distant metastasis was detected. The CT-guided percutaneous lung biopsy suggested poorly differentiated squamous cell carcinoma in primary. Bronchoscopy demonstrated no abnormality. On admission, routine laboratory studies including arterial blood gas analysis were normal. The pulmonary function test was normal. The patient received 2 cycles of neoadjuvant therapy with gemcitabine and cisplatin. The preoperative diagnosis was primary lung cancer, cT3N2M0, resectable IIIA stage.

**Fig. 2:**
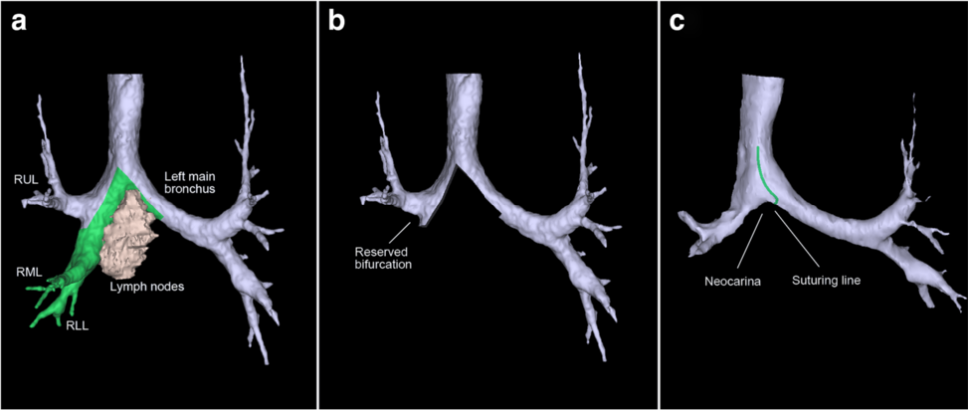
**a** The rapid prototyping image showed the relationship between the subcarinal lymph nodes and the airways (the *green* area showed the draft of resection part of the airways, including *left* main bronchus, carina, *right* main bronchus, and the bronchus intermedius). **b** The stimulated image of airways after resection. **c** The stimulated image of airways after carina reconstruction (the *green line* showed the expected suture line)

The patient was placed in a left lateral decubitus position. The double-lumen intubation was performed. The main incision was about 4 cm that located at the fourth intercostal space of the anterior axillary line. The assisted spot was located at the seventh intercostal space of the midaxillary line, and was used for introducing a 10 mm 30° thoracoscope. We completed the whole operation without visual access through the incision and without rib spreading (Additional file 1: Figure S1). We used electronic hook, curved suction apparatus, and other thoracoscopic instruments and equipment. Before surgery, radiology based rapid prototyping showed that the enlarged and fused subcarinal lymph nodes were close to the carina and the inner wall of bronchus (Fig. 2).

During the operation, the lung was first retracted anteriorly to mobilize the posterior hilum. Through detection, the subcarinal, the right posterior hilar and interlobar lymph nodes were found enlarged and fused together, highly attaching to the inner wall of left main bronchus, the carina, the inner wall of right main bronchus and the bronchus intermedius, which matched the preoperative evaluation. To achieve R0 resection, we decided to perform bilobectomy of right lower lobe and right middle lobe with carina resection and reconstruction.

**Fig. 3:**
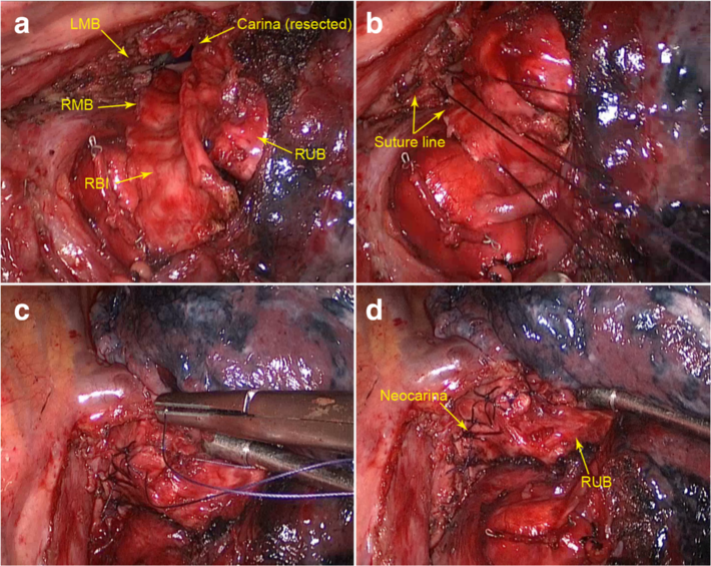
**a** The defect after the en-bloc resection of carina (the endotracheal tube was inserted into the *left* main bronchus). **b** The anastomosis between the *left* main bronchial semicircular defect and the semicircular wall of the *right* bronchus. **c** The closure of the carina. d The neocarina after reconstruction. LMB: *left* main bronchus; RMB: *right* main bronchus; RBI: *right* bronchus intemedius; RUB: *right upper* bronchus

After dividing the pulmonary ligament, the inferior pulmonary vein and the middle lobe vein were excised. The pulmonary arteries were excised in regular way through fissure. All the pulmonary vessels and fissure were manipulated by the mechanical staple. The specimen was removed after cutting off the distal bronchus intermedius by scissors. The carina, the inner walls of both left and right main bronchus were excised with the massive lymph nodes in an en-bloc fashion (Fig. 3a).

The reconstruction was made by 3–0 polene with running sutures. To decrease the tension, each one of the stitches was sutured for only 1 to 2 cm long. The closure of the cartilage portion was performed firstly. Then the left main bronchial semicircular defect was patched, using the residual semicircular wall of the right bronchus (Fig. 3b). The membranous portion was sutured at last. To avoid the airways instability, the spare cartilage tissue of the right bronchus was constrictively sutured onto the membranous portion of neocarina, and served as osseous support (Fig. 3c). The bifurcation between the right upper bronchus and the bronchus intermedius was used as the neocarina (Fig. 3d).

During the reconstruction, when the operator was resecting and suturing the bronchus or the carina, the anesthetist loosened the tube balloon and retracted the endotracheal tube above the carina, which spared the space for surgical manipulation. A little airway leakage in seconds was allowed and the patient would tolerate it well. Before the operator was knotting, the anesthetist inserted the tube back to its original position with the help of operator’s guide. During the reconstruction, no apnoea was used. After reconstruction, no air leakage was noted with a sustained airway pressure of 25 cmH_2_O. Coverage of anastomosis was made by the pericardium fat. Frozen sections of resection margins were negative. Intraoperative blood loss was about 220 mL, and operative time was about 225 min.

The postoperative course was uneventful. The chest tube was removed on postoperative day 4. The patient was discharged on postoperative day 7. The final pathologic examination confirmed the tumor invasion of the carina, and revealed metastases in the 4R, 7, 10R, 11R stations. Adjuvant chemotherapy using gemcitabine and cisplatin was administered for 4 cycles. Follow-up 6 months after surgery confirmed no stenosis and no signs of local recurrence by bronchoscopy and CT scan (Additional file 1: Figure S2).

## Discussion

The carina resection and reconstruction is indicated to the invasion of lower trachea or carina by malignance [1]. For non-small cell lung cancer, the overall survival of 5 years for carina resection ranges from 28.5 to 66.3 % [2–5]. The technique is various according to extent of invasion. In this case, the invasion is induced by the metastasized lymph nodes, which is a less common situation. During the operation, the invaded carina was resected and reconstructed by the natural bifurcation. This novel technique help the neocarina avoid neither a redundant suture nor an artificial reconstruction. Meanwhile, it reserves a functional carina and the stability of airways.

Before surgery, we used computed rapid prototyping to evaluate the invasion of the carina and the bronchus. The result showed the invaded wall was less than 50 % of airway circumference, which technically supported the performance of the reconstruction plan [6]. The rapid prototyping technique has just been used in thoracic surgery recently. Several researches report its advantage in pulmonary segmentectomy [7, 8]. In this case, the result of rapid prototyping was conformed to the intraoperative detection, which indicates the probability to use this technique in the surgery of central lung neoplasm.

## Conclusion

We consider that the surgical procedure described here is a new alternative strategy for carina resection and reconstruction in the similar situation. The minimally invasive method combined with rapid prototyping is safe and effective for this challenging operation.

## Additional file

Additional file 1: Figure S1. The surgical incisions. **Figure S2.** (A) Follow-up CT scan 6 months after surgery showed clear airways without recurrence. (B) Follow-up bronchoscopy 1 months after surgery showed the neocarina (the white arrow indicated the granulomatous hyperplasia). (C) The same bronchoscopy showed the normal distal right upper bronchus. (DOCX 1549 kb)
