# Supplementary material for: Two-port approached thoracoscopic carina reconstruction using natural bronchial bifurcation
Source: J Cardiothorac Surg. 2016 Oct 18;11:147. doi: 10.1186/s13019-016-0541-9 (PMC5070179; doi:10.1186/s13019-016-0541-9)
Supplement: Additional file 1: Figure S1. — The surgical incisions. Figure S2. (A) Follow-up CT scan 6 months after surgery showed clear airways without recurrence. (B) Follow-up bronchoscopy 1 months after surgery showed the neocarina (the white arrow indicated the granulomatous hyperplasia). (C) The same bronchoscopy showed the normal distal right upper bronchus. (DOCX 1549 kb) [file 13019_2016_541_MOESM1_ESM.docx]

## Supplements


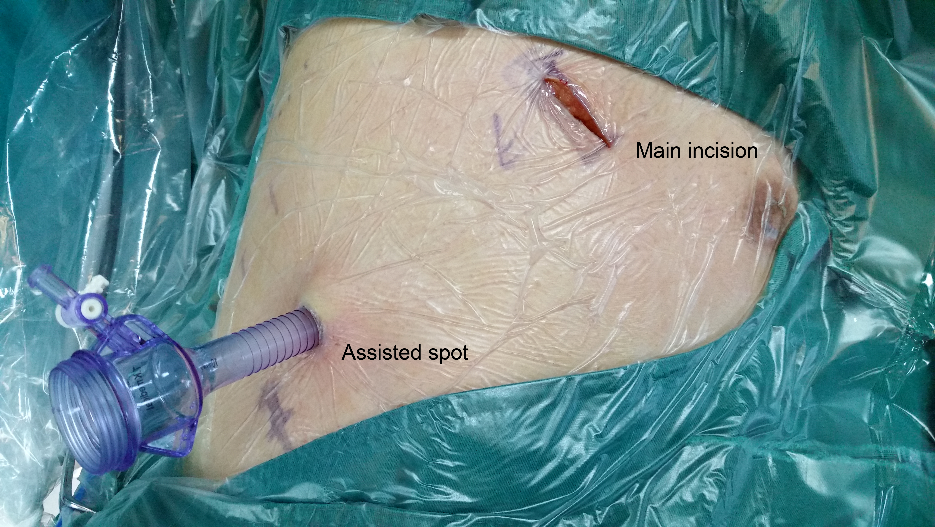


Figure 1 The surgical incisions.


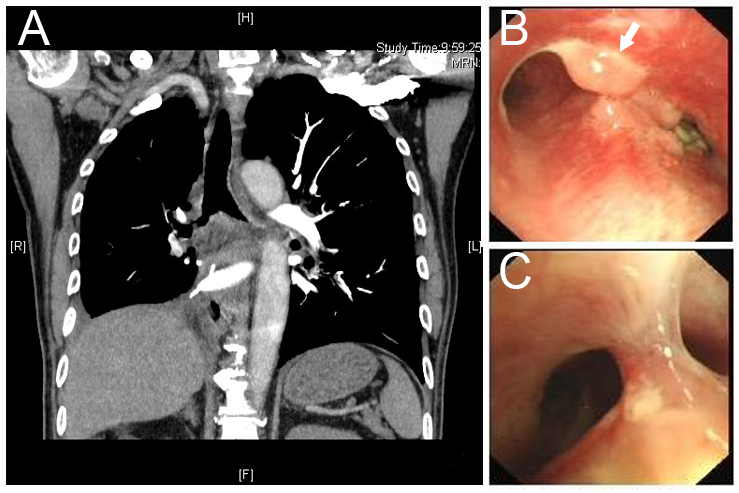


Figure 2 (A) Follow-up CT scan 6 months after surgery showed clear airways without recurrence. (B) Follow-up bronchoscopy 1 months after surgery showed the neocarina (the white arrow indicated the granulomatous hyperplasia). (C) The same bronchoscopy showed the normal distal right upper bronchus.
